# Temperature modulates systemic and central actions of thyroid hormones on BAT thermogenesis

**DOI:** 10.3389/fphys.2022.1017381

**Published:** 2022-11-18

**Authors:** Eva Rial-Pensado, Verónica Rivas-Limeres, Carmen Grijota-Martínez, Amanda Rodríguez-Díaz, Valentina Capelli, Olga Barca-Mayo, Rubén Nogueiras, Jens Mittag, Carlos Diéguez, Miguel López

**Affiliations:** ^1^ Department of Physiology, CIMUS, University of Santiago de Compostela- Instituto de Investigación Sanitaria, Santiago de Compostela, Spain; ^2^ CIBER Fisiopatología de la Obesidad y Nutrición (CIBERobn), Madrid, Spain; ^3^ Department of Cell Biology, Faculty of Biology, Complutense University, Madrid, Spain; ^4^ Alberto Sols Biomedical Research Institute (CSIC-UAM), Madrid, Spain; ^5^ Institute for Endocrinology and Diabetes—Molecular Endocrinology, Center of Brain Behavior and Metabolism CBBM, University of Lübeck, Lübeck, Germany

**Keywords:** thyroid hormones, temperature, thermogenesis, brown adipose tissue, hypothalamus, AMPK

## Abstract

Thyroid hormones (THs) play a major role regulating energy balance and brown adipose tissue (BAT) thermogenesis, as well as body temperature, as shown in hyperthyroid patients. However, the current landscape of preclinical thyroid hormone models is complex. For example, while rats become catabolic after TH administration, mice gain weight; so, these differences in species need to be analyzed in detail and specially whether temperature could be a factor. Here, we aimed to investigate the effect of environmental temperature on those actions. Rats were subcutaneously treated with L-thyroxine (T4) or stereotaxically within the ventromedial nucleus of the hypothalamus (VMH) with triiodothyronine (T3) and housed at 23°C, 4°C or 30°C; energy balance, BAT thermogenesis and AMP-activated protein kinase (AMPK) in the VMH were analyzed. Our data showed that the effect of both systemic T4 of central T3 on energy balance and BAT thermogenesis was dependent upon environmental temperature. This evidence is of interest in the design of experimental settings highlighting the species-specific metabolic actions of THs, and in understanding its physiological role in the adaptation to temperature.

## Introduction

Hyperthyroidism is a clinical syndrome in which overactive cells within the thyroid gland produce large amounts of THs, namely triiodothyronine (T3) and L-thyroxine (T4), leading to an excess of circulating free THs and increased metabolic rate. Notably, up to 85% of patients with hyperthyroidism exhibit weight loss despite increased food intake, both at mealtimes and between meals, with energy intake failing to meet the increased caloric demands associated with increased energy expenditure (López et al., 2013; Mullur et al., 2014; Capelli et al., 2021a). Usually, most of these effects have been attributed to the direct actions of THs on metabolically active tissues such as the liver, heart, skeletal muscle, and white (WAT) and brown (BAT) adipose tissue (Cannon and Nedergaard, 2010; López et al., 2013; Mullur et al., 2014; Johann et al., 2019; Capelli et al., 2021a).

Endotherms or warm-blooded animals maintain a constant body temperature independently of the environment. In contrast, ectotherms or cold-blooded animals regulate body temperature depending on external sources, such as sunlight or a heated rock surface (Grigg et al., 2022). The underlying reason for endothermy is mainly a more active metabolism and a lower thermodynamic efficiency (Hulbert and Else, 2004). Both sets of processes depend largely on the actions of THs, which lead to peripheral heat production and increased body temperature simultaneously; this suggests that THs might have played a crucial role in the development of endothermy (Cannon and Nedergaard, 2004; Silva, 2006; Warner and Mittag, 2012; López et al., 2013; Capelli et al., 2021a). Consequently, THs are critical for obligatory thermogenesis; that is, the heat production occurring as a byproduct of the metabolic rate (Cannon and Nedergaard, 2004; Silva, 2006; López et al., 2013). However, in addition to obligatory heat production, endothermic animals have developed facultative or adaptive thermogenesis, the production of heat on demand for a quick adaptation to changing environmental temperature (Cannon and Nedergaard, 2004; Silva, 2006; Warner and Mittag, 2012; López et al., 2013; Capelli et al., 2021a). While shivering is the earliest and most primitive response to cold, endothermic species have developed more efficient and long-term mechanisms of non-shivering facultative thermogenesis. The site and mechanism for facultative thermogenesis differ in the two main classes of current endothermic animals: birds and mammals. In birds, the main site for facultative thermogenesis is the skeletal muscle (Duchamp and Barre, 1993; Cannon and Nedergaard, 2010; Grigg et al., 2022), while in mammals, besides muscle (Cannon and Nedergaard, 2010; Johann et al., 2019), the major site is the BAT (Cannon and Nedergaard, 2004; Whittle et al., 2011; Villarroya and Vidal-Puig, 2013; Contreras et al., 2015; Villarroya et al., 2017).

THs are major regulators of thermogenesis by direct actions on brown adipocytes but also by central mechanisms involving the sympathetic nervous system (SNS)-mediated activation of BAT (Nedergaard et al., 1997; Cannon and Nedergaard, 2004; Silva, 2006; Sjogren et al., 2007; Cannon and Nedergaard, 2010; Warner and Mittag, 2012; López et al., 2013; Mullur et al., 2014; Capelli et al., 2021a). However, whether and how those effects are affected by environmental temperature is currently unclear. This is of interest not only in terms of evolutionary adaptation to cold and warm habitats (Grigg et al., 2022), but it also has clinical importance, for example, in the understanding and management of THs-related syndromes, such as hypothyroidism and hyperthyroidism, which are characterized by cold and heat intolerance, respectively (Silva, 2006; Warner and Mittag, 2012; López et al., 2013; Capelli et al., 2021a). Thus, the aim of this study has been to investigate the effect of ambient temperature on THs actions on energy balance and BAT thermogenesis in rats.

## Materials and methods

### Animals

Adult male Sprague-Dawley rats (8–10 weeks old, 200–250 g; CEBEGA USC, Santiago de Compostela, Spain) were housed with an artificial 12-h light (8:00–20:00)/12-h dark cycle, under controlled temperature (23°C) and humidity (50% ± 5%) conditions. Before starting the experimental procedure, the animals underwent a 7-day period of acclimatization to the facility and to the handling procedure under non-stressful conditions. Animals had free access to a standard laboratory chow diet (STD, SAFE A04: 3,1% fat, 59,9% carbohydrates, 16,1% proteins, 2.791 kcal/g; *Scientific Animal Food & Engineering*; Nantes, France) and tap water. All experiments and procedures were performed in agreement with International Law on Animal Experimentation and USC Ethical Committee (project ID and 15012/2020/10).

### Induction of hyperthyroidism

Hyperthyroidism was induced by chronic subcutaneous (SC) administration of L-thyroxine (T4, 100 µg/day, dissolved in 200 µl of saline; Sigma-Aldrich; St Louis, MO, United States) for a period of 4 weeks (28 days) as previously described (López et al., 2010; Varela et al., 2012; Martinez-Sanchez et al., 2017a; Martínez-Sánchez et al., 2017). Euthyroid (control) rats were treated with vehicle (saline). Food intake and body weight were weighted daily with a precision scale.

### Stereotaxic microinjection of T3

Rats were placed in a stereotaxic frame (*David Kopf Instruments;* Tujunga, CA, United States) under ketamine/xylazine anesthesia. Nuclei-specific injections were delivered *via* a permanent 28-gauge stainless steel cannula (*Plastics One*, Roanoke, VA, United States) inserted bilaterally in the ventromedial nucleus of the hypothalamus (VMH) following stereotaxic coordinates: −2.8 mm posterior to the bregma, ±0.6 mm lateral to bregma and 10.1 mm deep from the skull. A catheter tube was connected from each infusion cannula to an osmotic minipump flow moderator (Model 1007D; *Alzet Osmotic Pumps*, Cupertino, CA, United States). These pumps had a flow rate of 0.5 μl/h during 7 days of treatment. T3 was given at 4 ng/day (in saline +1 mM NaOH) and vehicle (saline +1 mM NaOH) used as control. The selection of these doses was based on previous reports (López et al., 2010; Martinez-Sanchez et al., 2017a; Martínez-Sánchez et al., 2017). The osmotic minipumps were inserted in a subcutaneous pocket on the interscapular surface created using blunt dissection (Martins et al., 2016; Martinez-Sanchez et al., 2017a; Martínez-Sánchez et al., 2017; Rial-Pensado et al., 2022). The correct position of the cannuale was verified by histological examination of coronal sections of the brains, as shown (López et al., 2010; Martínez de Morentin et al., 2014; Martins et al., 2016; Martínez-Sánchez et al., 2017).

### Temperature challenge

For systemic hyperthyroidism, on day 28, animals were split in three groups: **1)** the first set of rats was maintained at 23°C, **2)** a second set was kept at 4°C, and **3)** a third set was kept at 30°C. In all the cases, rats were subjected to the new housing temperatures for 4 days (day 28–day 31), based on former studies (López et al., 2010; Alvarez-Crespo et al., 2016; Martinez-Sanchez et al., 2017a; Martinez-Sanchez et al., 2017b; Johann et al., 2019; Capelli et al., 2021b); during that time, rats were also T4-treated. For VMH T3 treatments rats were split into the same three experimental settings from the day of the surgery and the beginning of the treatment; they were maintained at the three different housing temperatures (23°C, 4°C and 30°C) for 7 days.

### Core body temperature and brown adipose tissue temperature measurements

Core body temperature was measured using a rectal probe connected to digital thermometer (*BAT-12 Microprobe-Thermometer; Physitemp*; Clifton, NJ, United States). Skin temperature surrounding BAT (which was shaved some days before for adaptation), was recorded with an infrared camera (*B335: Compact-Infrared-Thermal-Imaging-Camera*; *FLIR*; West Malling, Kent, United Kingdom) and analyzed with a specific software package (*FLIR-Tools-Software, FLIR*; West Malling, Kent, United Kingdom), as previously shown (Martínez de Morentin et al., 2012; Martínez de Morentin et al., 2014; Martins et al., 2016; Milbank et al., 2021; Oelkrug and Mittag, 2021; Urisarri et al., 2021; Seoane-Collazo et al., 2022). For each image, the average temperatures were calculated as the average of 2-3 pictures/animal. Animals were previously used to handling to avoid stress-induced BAT thermogenesis.

### Sample processing

Animals were sacrificed on day 31 (T4 SC) or 7 (T3 VMH) by cervical dislocation. From each animal, the whole hypothalamus (for RIA) or the VMH (dissected from the whole hypothalamus, for western blotting) were extracted, as well as the BAT. The blood of the animals was collected in 1.5 ml tubes and centrifuged for 15 min at 2,000 rpm to separate the serum. Samples were stored at −80°C until further processing.

### Radioimmunoassay

Total T4 and T3 determination in serum and tissues was performed as previously described (Capelli et al., 2021b). To estimate the yield of the TH extraction, small amounts of ^125^I-T3 and ^125^I-T4 were added as internal tracers in serum and tissue initial homogenates. The detection range for the RIA assay was 0.4–100 pg T3/tube and 2.5–320 pg T4/tube.

### Real-time quantitative RT-PCR

Real-time PCR (*TaqMan; Applied Biosystems;* Foster City, CA, United States) was performed using specific primers and probes [*Hprt*: Fw: 5′- AGC CGA CCG GTT CTG TCA-3′; Rv: 5′- GGT CAT AAC CTG GTT CAT CAT CAC -3′; Probe:5′- (FAM)-C GAC CCT CAG TCC CAG CGT CGT GAT- (TAM)3′; β3 adrenergic receptor, *Adrb3*: Rn01478698_g1 Adrb3 *Thermo Fisher Scientific*; Karlsruhe, Germany], as previously described (López et al., 2010; Martínez de Morentin et al., 2014; Seoane-Collazo et al., 2019; Milbank et al., 2021; Seoane-Collazo et al., 2021). Values were expressed in relation to hypoxanthine-guanine phosphoribosyl-transferase (*Hprt*) levels.

### Western blotting

Protein lysates from VMH and BAT were homogenized in lysis buffer and subjected to SDS-PAGE, electrotransferred to polyvinylidene difluoride membranes (PVDF; *Millipore*; Billerica, MA, United States) with a semidry blotter and probed with antibodies against phopho-AMP-activated protein kinase alpha (pAMPKα) (Thr172), uncoupling protein 1 (UCP1) (*Abcam*; Cambridge, United Kingdom), α-tubulin or β-actin (*Sigma-Aldrich*; St. Louis, MO, United States), as previously described (López et al., 2010; Martínez de Morentin et al., 2014; Martins et al., 2016; Seoane-Collazo et al., 2019; Capelli et al., 2021b; Milbank et al., 2021; Seoane-Collazo et al., 2021). Each membrane was incubated with the corresponding secondary antibody: anti-mouse or anti-rabbit (all from *DAKO*; Glostrup, Denmark). Autoradiographic films were scanned, and the bands signal was quantified by densitometry using *ImageJ-1.33 software* (*NIH*; Bethesda, MD, United States). Values were expressed in relation to β-actin (VMH) or α-tubulin (BAT). Representative images for all proteins are shown. In all the Figures showing images of gels, all the bands for each picture always come from the same gel; however, they may be spliced for clarity.

### Statistical analysis

Statistical analysis was conducted using *GraphPad Prism 8 Software* (*GraphPad Software*; La Jolla, CA, United States). Data are expressed as MEAN ± SEM as a percentage of the controls (vehicle-treated mice) when relativized. Statistical significance was determined by Mixed effect analysis (for time course treatments), Student’s t-test (when two groups were compared) or one-way ANOVA followed by Tukey test (when more than two groups were compared in Supplemental information graphs). *p* < 0.05 was considered significant.

## Results

### Peripherally induced hyperthyroidism impacts body weight in a temperature-dependent fashion

**FIGURE 1:**
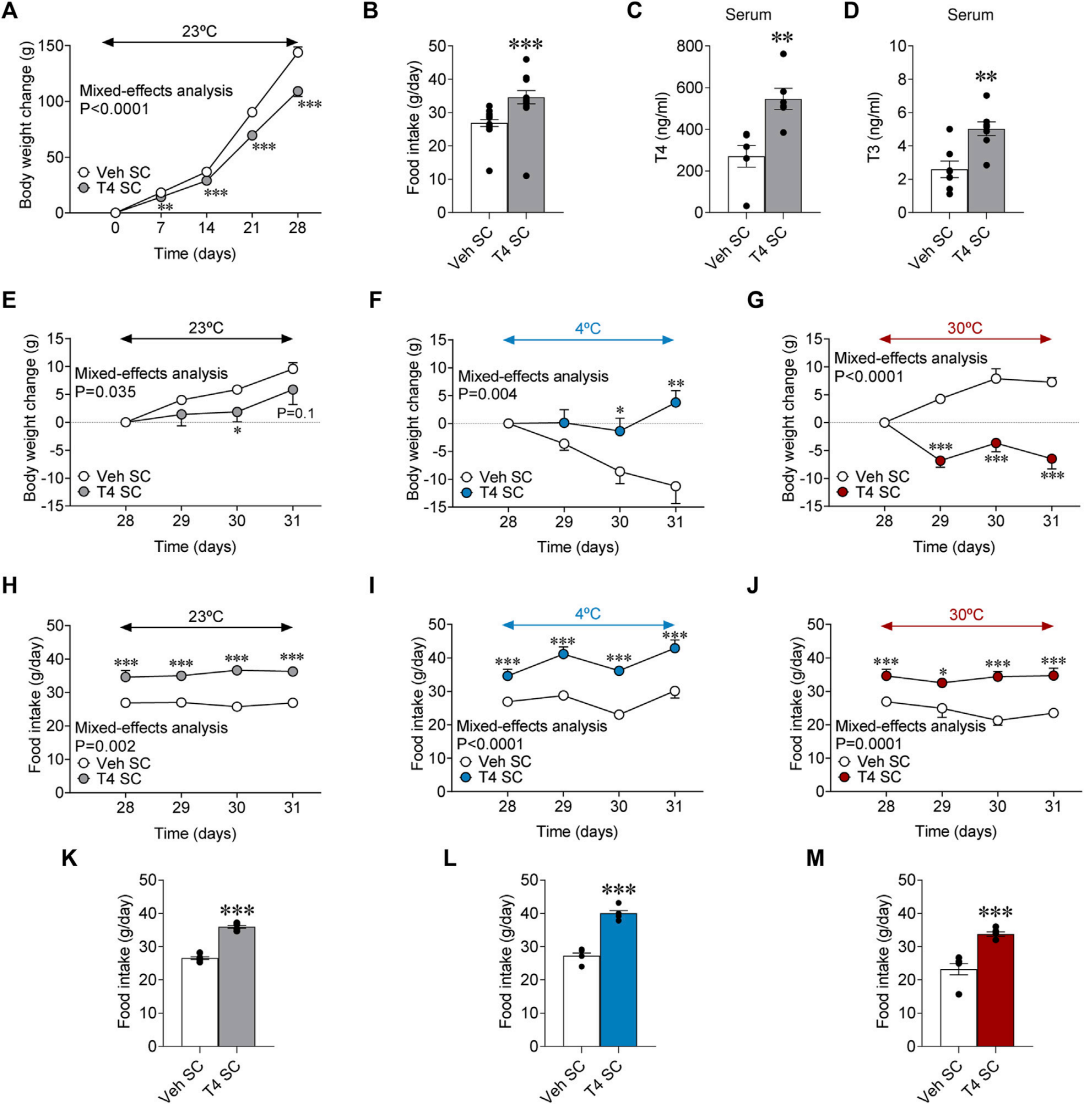
Effects of temperature on energy balance in hyperthyroid rats **(A)** Body weight change, **(B)** daily food intake, **(C)** serum T4 and **(D)** serum T3 in euthyroid and hyperthyroid rats [peripherally treated (SC) with vehicle or T4]. **(E–G)** Body weight change, **(H,I)** food intake, and **(K,L)** daily food intake of euthyroid, and hyperthyroid rats housed at 23°C **(E,H,K)**, 4°C **(F,I,L)** or 30°C **(G,J,M)**. Data are represented as MEAN ± SEM. *n* = 23–24 **(A,B)** and 6–8 rats/group **(C–M)**. Statistical significance was determined by Mixed effect analysis or Student’s *t*-test. *p < 0.05, **p < 0.01, ***p < 0.001 vs. vehicle SC.

SC treatment with T4 reduced weight gain [**Day 0 =** vehicle SC: 243.92 ± 3.90 g vs. T4 SC: 247.71 ± 2.88, non-significant. **Day 28 =** vehicle SC: 387.79 ± 4.77 g vs. T4 SC: 356.92 ± 4.17, *p* < 0.001] and promoted hyperphagia (Figures 1A,B), as well as a rise in the circulating levels or T4 and T3 (Figures 1C,D), as formerly described (López et al., 2010; Varela et al., 2012; Martinez-Sanchez et al., 2017a; Martínez-Sánchez et al., 2017). Next, we aimed to investigate the effect of housing temperature on the impact of peripherally induced hyperthyroidism on body weight and feeding. All split euthyroid [**23°C**: 398.86 ± 9.85 g; **4°C**: 380.88 ± 3.91 g; **30°C**: 385.43 ± 6.74 g] and hyperthyroid [**23°C**: 352.50 ± 7.49 g; **4°C**: 355.75 ± 8.05 g; **30°C**: 360.50 ± 5.31 g] groups had similar body weights at day 28. Our data showed that while hyperthyroid rats maintained at 23°C continued to reduce their weight gain (Figure 1E) and their absolute body weight [**Day 31 =** vehicle SC: 411.0 ± 10.42 g vs. T4 SC: 359.13 ± 8.77, *p* < 0.001**]**, despite hyperphagia (Figures 1H,K), hyperthyroid rats kept at 4°C increased their body weight gain when compared to euthyroid controls, which reduced it (Figure 1F) and displayed hyperphagia (Figures 1I,L). On the other hand, hyperthyroid rats kept at 30°C −a temperature considered thermoneutral for rodents (Johann et al., 2019; Milbank et al., 2021)− exhibited marked body weight loss (Figure 1G), and decreased their absolute body weight [**Day 31 =** vehicle SC: 386.75 ± 8.79 g vs. T4 SC: 355.00 ± 7.08, *p* < 0.01] regardless of the increased food intake (Figures 1J,M).

### Peripherally induced hyperthyroidism increased hypothalamic thyroid hormone levels independently of temperature

**FIGURE 2:**
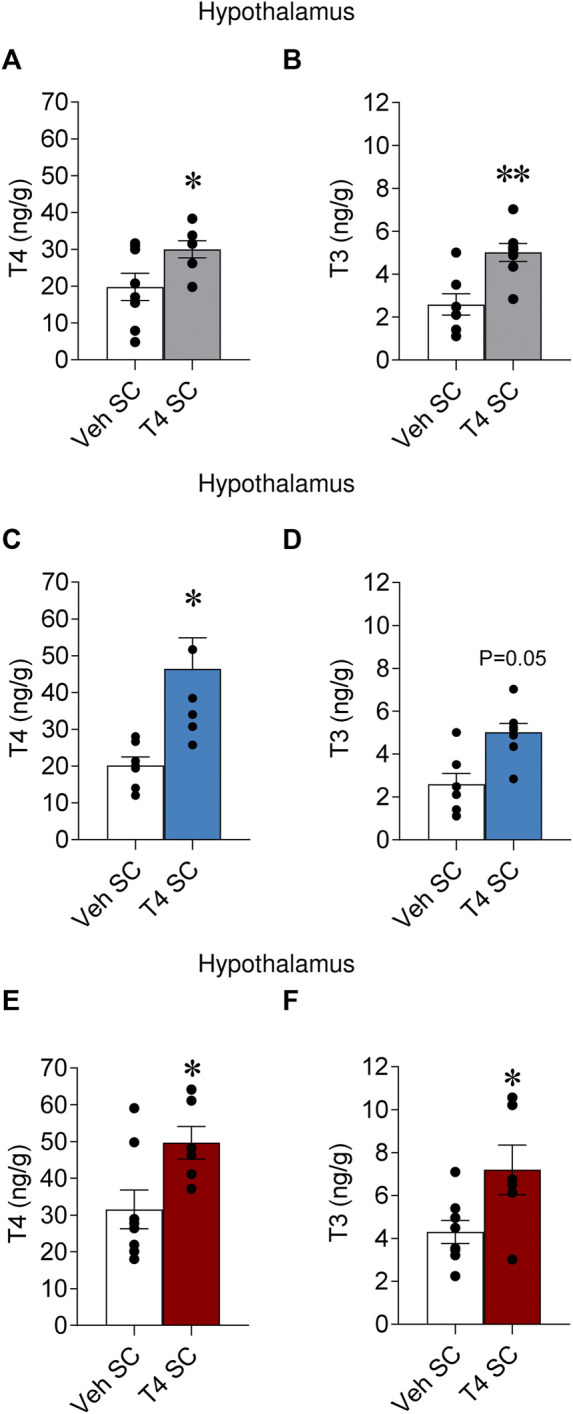
Effects of temperature on hypothalamic TH levels in hyperthyroid rats Hypothalamic T4 and T3 levels of euthyroid and hyperthyroid rats housed at 23°C **(A,B)**, 4°C **(C,D),** and 30°C **(E,F)**. Data are represented as MEAN ± SEM. *n* = 6–8 rats/group. Statistical significance was determined by Student’s *t*-test. *p < 0.05, **p < 0.01*.* Vehicle SC

Systemic hyperthyroidism induced an elevation of TH levels in the hypothalamus (Capelli et al., 2021b); however, it is unclear whether temperature may affect that effect. Thus, we assayed T4 and T3 levels in the hypothalamus of hyperthyroid rats kept at 23°C, 4°C and 30°C. Our data showed that both T4 and T3 were increased **(** Figures 2A–F; non-significant trend for T3 at 4°C) in the hypothalamus of hyperthyroid rats. This suggested that ambient temperature does not impact the hyperthyroidism-induced increase of hypothalamic TH levels.

### Peripherally induced hyperthyroidism increased brown adipose tissue thermogenesis in a temperature-dependent fashion

**FIGURE 3:**
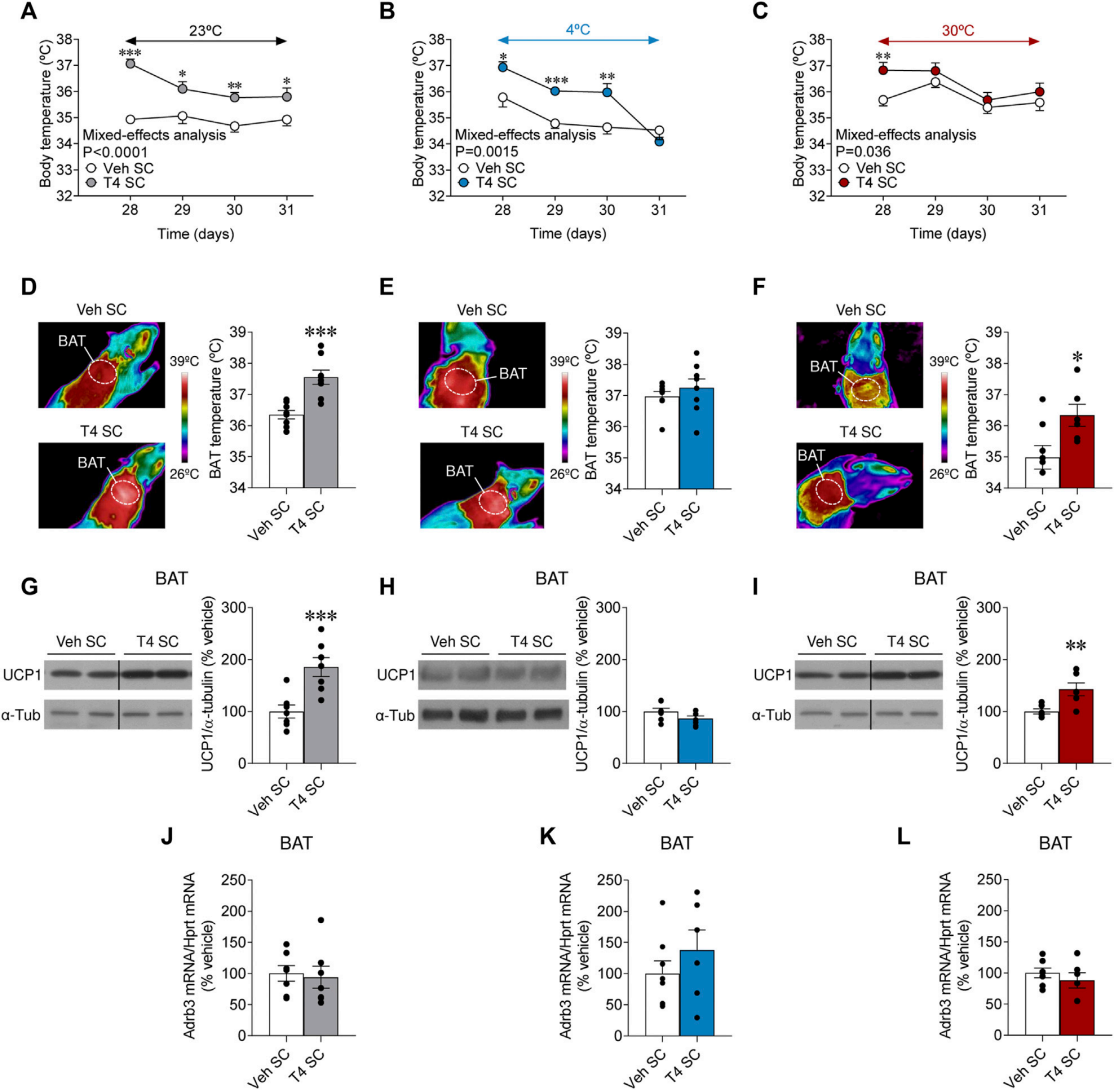
Effects of temperature on BAT thermogenesis in hyperthyroid rats **(A–C)** Body temperature, **(D–F)** BAT temperature, **(G–I)** UCP1 protein levels in BAT, and **(J–L)** *Adrb3* mRNA levels in BAT of euthyroid and hyperthyroid rats housed at 23°C **(A,D,G,J)**, 4°C **(B,E,H,K)** or 30°C **(C,F,I,L)**. Data are represented as MEAN ± SEM. *n* = 6–8 rats/group. Statistical significance was determined by Mixed effect analysis or Student’s *t*-test. *p < 0.05, **p < 0.01, ***p < 0.001 vs. vehicle SC.

THs are major regulators of body temperature and thermogenesis (López et al., 2010; Warner et al., 2013; Alvarez-Crespo et al., 2016; Martinez-Sanchez et al., 2017a; Martínez-Sánchez et al., 2017; Capelli et al., 2021a); however the peripheral actions of THs on the thermogenic mechanisms, and specially of BAT, have been a matter of discrepancy in the literature with apparent differences in mice versus rats (López et al., 2010; Warner et al., 2013; Dittner et al., 2019; Johann et al., 2019; Capelli et al., 2021a). Our results showed that hyperthyroid rats exhibited further increased body temperature at 23°C and 4°C but not at 30°C (Figures 3A–C). As expected, hyperthyroid rats kept at 4°C were initially able to defend body temperature better than euthyroid controls through the course of the experiment, although after 4 days at cold exposure they were no longer different than controls (Figure 3B). On the other hand, when exposed at 30°C, hyperthyroid rats only showed elevated body temperature the first day (day 28). Still, they displayed similar levels from the remaining exposure time compared to euthyroid controls (Figure 3C). BAT temperature was increased in hyperthyroid rats maintained at 23°C and 30°C when compared with their respective controls, but not in cold-exposed hyperthyroid rats, presumably due to the hyperactivation of the tissue by the cold in control animals as well (Figure 3D,E). Contrary, basal BAT temperature was lower in euthyroid rats housed at 30°C (Figure 3F). This evidence suggested that cold stimulation, but not thermoneutrality, led to BAT activation, which cannot be further augmented by T4.

To gain further insight into the molecular mechanisms mediating hyperthyroid-induced body temperature, we investigated the effect of temperature on UCP1 protein levels and the mRNA expression of *Adrb3* in BAT. Firstly, we examined the effect of temperature in euthyroid (control) rats. While, as known (Cannon and Nedergaard, 2004), UCP1 protein content was augmented in the brown fat of cold-exposed animals, when compared to both rats at room temperature and 30°C (Supplementary Figure S1A), *Adrb3* mRNA expression was reduced in the same experimental group (Supplementary Figure S1B), as described (Bengtsson et al., 1996). Secondly, we assayed the effect of temperature and T4 on the same parameters. UCP1 protein levels in BAT were increased in hyperthyroid rats at 23°C and 30°C but not at 4°C (Figures 3G–I), likely because cold stimulation already increased the UCP1 protein content (Supplementary Figure S1A). *Adrb3* mRNA levels in BAT were not affected by hyperthyroidism at any temperature (Figures 3J–L). Overall, this evidence demonstrates that peripherally induced hyperthyroidism increased BAT thermogenesis in a temperature-dependent fashion.

### Central hyperthyroidism decreases body weight in a temperature-dependent fashion

**FIGURE 4:**
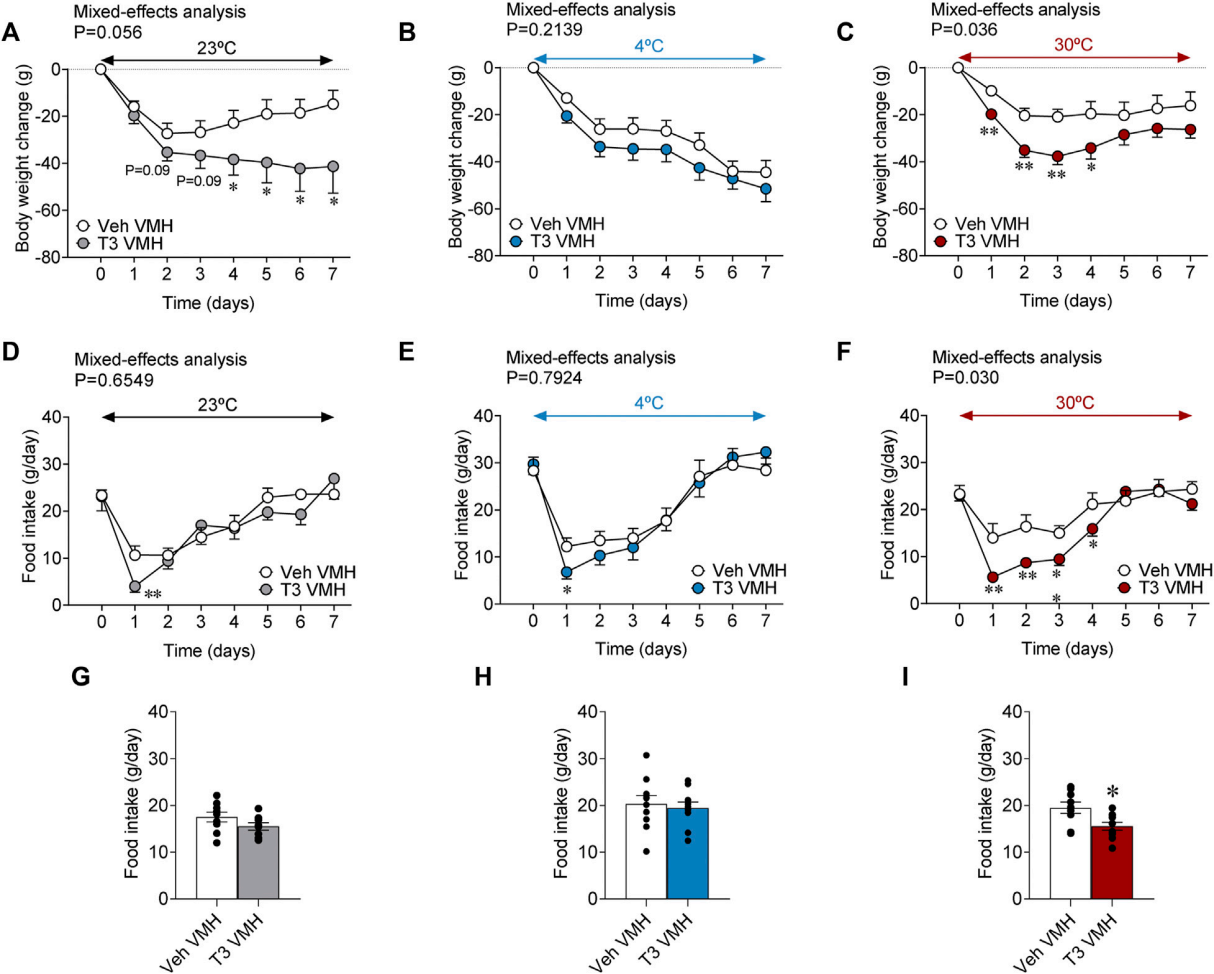
Effects of temperature on energy balance in rats centrally treated with T3 **(A–C)** Body weight change, **(D–F)** food intake, and **(G–I)** daily food intake of rats stereotaxically treated with vehicle or T3 housed at 23°C **(A,D,G)**, 4°C **(B,E,H)** or 30°C **(C,F,I)**. Data are represented as MEAN ± SEM. *n* = 9–11 rats/group. Statistical significance was determined by Mixed effect analysis or Student’s t-test. *p < 0.05, **p < 0.01 vs. vehicle VMH.

Current evidence has emerged about the central role of THs in the regulation of whole-body metabolism (Sjogren et al., 2007; Klieverik et al., 2009a; Klieverik et al., 2009b; Fliers et al., 2010; Kalsbeek et al., 2010; López et al., 2010; Yi et al., 2010; Mittag et al., 2013; de Vries et al., 2014; Fliers et al., 2014; Kalsbeek et al., 2014; Alvarez-Crespo et al., 2016; Martinez-Sanchez et al., 2017a; Martínez-Sánchez et al., 2017; Herrmann et al., 2020; Capelli et al., 2021a). In particular, the central actions of T3 within the VMH are critical in the regulation of thyroid-induced thermogenesis (López et al., 2010; Martinez-Sanchez et al., 2017a; Martínez-Sánchez et al., 2017; Capelli et al., 2021a). However, whether those actions are affected by ambient temperature is unknown. Our results showed that T3 stereotaxically given within the VMH induced time-dependent weight loss when rats were kept at 23°C (long-term) and 30°C (initial and transient) but not at 4°C (a condition where both groups decreased body weight) when compared with vehicle-treated controls (Figures 4A–C). All split groups had similar body weights at day 0 [**23°C**: 440.65 ± 10.34 g; **4°C**: 450.40 ± 5.13 g; **30°C**: 455.10 ± 6.84 g]. Of note, while feeding was not affected by central T3 administration at 23°C and 4°C, it elicited hypophagia at 30°C (Figures 4D–I), which is somewhat expected as a mechanism to prevent a further increase, food mediated, in temperature.

### Central hyperthyroidism increased brown adipose tissue uncoupling protein 1 and inhibits hypothalamic AMP-activated protein kinase in a temperature-dependent fashion

**FIGURE 5:**
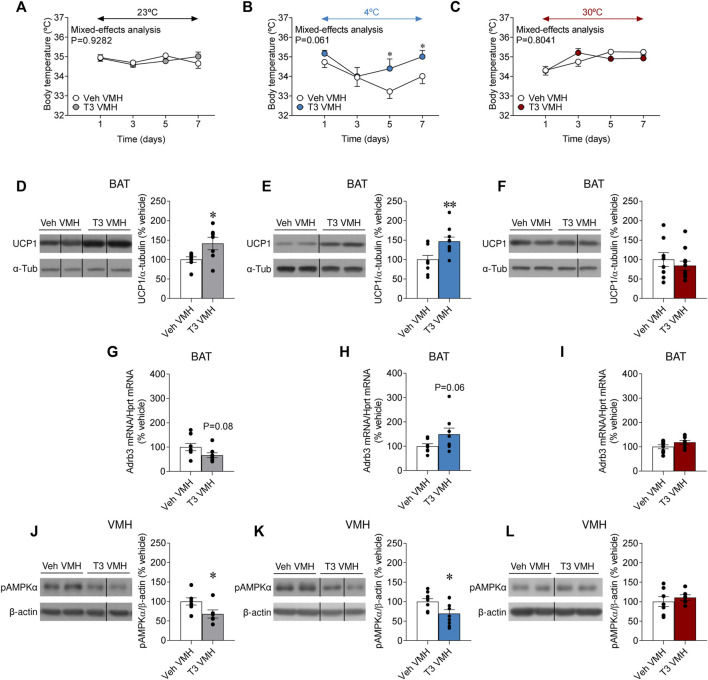
Effects of temperature on BAT thermogenesis and hypothalamic AMPK in rats centrally treated with T3 **(A–C)** Body weight change, **(D–F)** UCP1 protein levels in BAT, **(G–I)** *Adrb3* mRNA levels in BAT, and **(J–L)** pAMPKα protein levels in the VMH of rats stereotaxically treated with the vehicle of T3 housed at 23°C **(A,D,G,J)**, 4°C **(B,E,H,K)** or 30°C **(C,F,I,L)**. Data are represented as MEAN ± SEM. *n* = 6–11 rats/group. Statistical significance was determined by Mixed effect analysis or Student’s t-test *p < 0.05, **p < 0.01 vs. vehicle VMH.

Central treatment with T3 has been demonstrated to rise body temperature (Alvarez-Crespo et al., 2016). However, no data have addressed whether that action depends upon housing conditions. Firstly, we examined the effect of temperature in vehicle-treated rats. Similarly to euthyroid animals (also vehicle-treated) in the systemic T4 model (Supplementary Figure S1A), UCP1 protein content was augmented in the brown fat of cold-exposed animals, when compared to both rats at room temperature and 30°C (Supplementary Figure S2A), whereas, again, *Adrb3* mRNA expression was reduced in the same experimental group (Supplementary Figure S2B) (Bengtsson et al., 1996). Moreover, our results showed that T3 in the VMH allowed to defend body temperature when rats were kept at 4°C, but no differences were found when rats were housed at 23°C and 30°C (Figures 5A–C). Notably, despite the lack of effect of VMH T3 on body temperature at 23°C, it increased UCP1 protein levels in the BAT (Figure 5D). When given at 4°C, VMH T3 could also induce BAT UCP1 levels (Figure 5E), an effect that was missed at 30°C (Figure 5F). *Adrb3* mRNA levels in BAT where not deeply affected by central T3 or housing temperature, with only non-significant trends to be decreased at 23°C and increasing at 4°C (Figures 5G–I).

To gain further insight into the hypothalamic mechanisms mediating the effects on BAT, we analyzed AMPK in the VMH, a canonical regulator of BAT thermogenesis (López et al., 2016; Lopez, 2018, 2022). In keeping with the UCP1 BAT results, VMH T3 decreased the activation of AMPK (as demonstrated by decreased pAMPKα levels) in the VMH of rats kept at 23°C and four°C, but not at 30°C (Figures 5J–L). Overall, this evidence demonstrates that central hyperthyroidism increased BAT thermogenic program in a temperature-dependent fashion.

## Discussion

THs are major regulators of energy balance. They act both at peripheral and central levels and modulate BAT function and the expression of key thermogenic markers (López et al., 2010; Warner et al., 2013; Mullur et al., 2014; Alvarez-Crespo et al., 2016; Martinez-Sanchez et al., 2017a; Martínez-Sánchez et al., 2017; Capelli et al., 2021a). The physiological role of peripheral TH action on BAT has been controversial (Dittner et al., 2019; Johann et al., 2019; Capelli et al., 2021b), presumably also caused by minor yet important species idiosyncrasies. For instance, recent data in mice have shown that at thermoneutrality (30°C), T4 administration leads to a marked increase in metabolism and body temperature that is UCP1-independent (Dittner et al., 2019), while our results show that peripheral administration of T4 to rats increased body temperature at 23°C and 4°C, but not at 30°C. Notably, increased body temperature was associated with T4-induced expression of UCP1 in brown fat at 23°C and 30°C, but not at 4°C, a setting in which basal UCP1 is already highly increased, presumably masking any additional increase caused by T4.

Our data indicate that increased BAT temperature upon T4 administration is dependent on UCP1 activity in rats. There are several possible reasons for these discrepancies with the T4 model in mice. Firstly, rats and mice show differential responses to THs regarding energy balance. For example, in rats THs elicit a robust catabolic phenotype like humans, characterized by a massively reduced weight gain and lower adiposity (regardless of hyperphagia) (López et al., 2010; Varela et al., 2012; Martinez-Sanchez et al., 2017a); in mice THs (both T3 and T4) promote weight gain and lean mass accretion, possibly due to enhanced growing (Dittner et al., 2019; Johann et al., 2019; Capelli et al., 2021b). This increased size subsequently promotes a decrease in the surface-to-volume ratio (S/V), which is one of the most important critical factors modulating heat dissipation, and the reason why larger animals defend their temperature better because they display lower S/V (Cannon and Nedergaard, 2004; Silva, 2006; Grigg et al., 2022). It would be conceivable that the TH-induced increase in body dimensions may account for better S/V, leading to diminished heat losses and making BAT thermogenesis more dispensable. In this sense, we have recently reported that after the administration of TH in drinking water to mice, BAT temperature remains unchanged, despite increased body temperature, accounting for alternative heat sources (Capelli et al., 2021b). Secondly, the fact that TH-treated mice increased lean mass (Dittner et al., 2019; Johann et al., 2019) makes the involvement of muscle-based thermogenic mechanism more likely, as we have recently demonstrated (Johann et al., 2019), making again BAT thermogenesis less essential. Thirdly, the treatment length is also relevant; in former studies, mice were treated with T3 or T4 in the range of 14–21 days, while in our rat model, the treatment lasted for 31 days. This also applies to cold stimuli, which were acute in the aforementioned study (1 h) (Dittner et al., 2019), while more chronic in our setting (4 days).

Emerging evidence obtained in the last 15 years has demonstrated that the central actions of THs on thermogenesis are as relevant as their peripheral effect on brown adipocytes (López et al., 2010; Alvarez-Crespo et al., 2016; Martinez-Sanchez et al., 2017a; Martínez-Sánchez et al., 2017; Capelli et al., 2021a). To obtain better insight into this physiological mechanism, we examined the effect of chronic central T3 on energy homeostasis in the same three temperature housing settings (23°C, 4°C and 30°C). T3 was given in the VMH, a key nucleus regulating sympathetic tone on BAT *via* inhibition of AMPK (López et al., 2010; Martinez-Sanchez et al., 2017a; Martínez-Sánchez et al., 2017; Capelli et al., 2021a). It is important to highlight that since subcutaneous minipumps for the VMH administration were placed in the interscapular area, the analysis of BAT temperature by thermography was technically not feasible in this experimental model. As shown, VMH T3 induced marked feeding-independent weigh loss at 23°C and 30°C. At 4°C both groups of rats reduced their body weight, despite hyperphagia (compare basal levels in Figures 4D–F). Our evidence also showed that VMH T3 promoted increased UCP1 protein levels in the BAT in association with decreased AMPK activation in the VMH at 23°C and 4°C, but not at 30°C. These data indicate that the central action of TH in the VMH promotes BAT thermogenesis depending on environmental temperature. At 4°C, the inhibition of AMPK signaling in the VMH promoted increased BAT UCP1, associated with increased body temperature, suggesting that the central action of T3 helps to defend temperature upon cold stimulation. That effect was not observed at 23°C; the reasons for this are unclear, but one possibility is that T3-treated rats were already heat-stressed and trying to dissipate heat to avoid hyperthermia. This is supported by the fact that *Adrb3* mRNA expression in the BAT tends to decrease, maybe to reduce sympathetic tone. Further work analyzing the heat-dissipating mechanism, for example, by regulating vasodilation and vasoconstriction at the tail level (Warner et al., 2013), as well as their whole-body insulating response to changing temperatures, will help to clarify this issue. Notably, at 30°C, VMH T3 did not elicit any change in either hypothalamic pAMPK or BAT UCP1. These data recapitulate our recent evidence using the drinking T3 mouse model (Johann et al., 2019), indicating that in this thermoneutral scenario, the engagement of the AMPK(VMH)-SNS-BAT is not needed, and an upstream thermoregulatory mechanism may be inhibiting the activation of this axis.

Altogether, the presented and already reported evidence (López et al., 2010; Alvarez-Crespo et al., 2016; Martinez-Sanchez et al., 2017a; Martinez-Sanchez et al., 2017b; Johann et al., 2019; Capelli et al., 2021b) indicates that rats and mice are fundamentally different in the thermogenic response to TH: in rats systemic (and central) THs act within the VMH *via* decreased pAMPK to sympathetically-activate BAT (López et al., 2010; Varela et al., 2012; Martinez-Sanchez et al., 2017a; Martínez-Sánchez et al., 2017). At 4°C, cold stimulation led to massive BAT activation, which cannot be further augmented by T4 (hence no difference in BAT temperature is seen anymore, as it is already elevated). However, at 30°C, there is some higher inhibition of the VMH, presumably to avoid hyperthermia, which prevents T3 from affecting pAMPK and consequently, BAT. In contrast, in mice, systemic TH does not activate BAT thermogenesis (despite increased BAT recruitment as evidenced by elevated UCP1 levels) while still increasing body temperature (Johann et al., 2019; Capelli et al., 2021b). Therefore, our data indicated that systemic TH might cause pyrexia (fever) only in mice (Dittner et al., 2019), but the situation may be more complex in rats. Besides the effects on body weight (reduced in rats and enhanced in mice), these results suggest that conclusions obtained in rodent models − particularly in mice − need to be taken with care when translating to humans. Nevertheless, in keeping with our data, it has been recently reported, by using a prospective cohort study, that cold-induced thermogenesis is not increased in patients with overt hyperthyroidism (Maushart et al., 2022); this suggests that the evolutionary response to cold adaptation may not differ between species. In this regard, it will be interesting to address whether the effect of temperature on TH action depends on other factors such as gender or body mass, for example, using rats fed a high-fat diet. Preliminary data obtained by our group indicate that the central effects of T3 are maintained in obese rats (data not shown), but further work will be needed to address this issue.

In summary, our data show that environmental temperature modulates systemic and central actions of thyroid hormones on BAT thermogenesis and energy balance in a complex manner, integrating the hormone’s actions with the need for thermogenesis and the danger of hyperthermia. This is of interest for the design of experimental settings to investigate the hormone’s metabolic and thermoregulatory actions, including hyper- and hypothyroidism, and in the understanding of its physiological and evolutionary role in the adaptation to environmental temperature.

## Data Availability

The raw data supporting the conclusion of this article will be made available by the authors, without undue reservation.
