# Supplementary material for: Temperature modulates systemic and central actions of thyroid hormones on BAT thermogenesis
Source: Front Physiol. 2022 Nov 18;13:1017381. doi: 10.3389/fphys.2022.1017381 (PMC9716276; doi:10.3389/fphys.2022.1017381)
Supplement: Supplementary file 1 [file DataSheet1.pdf]

A

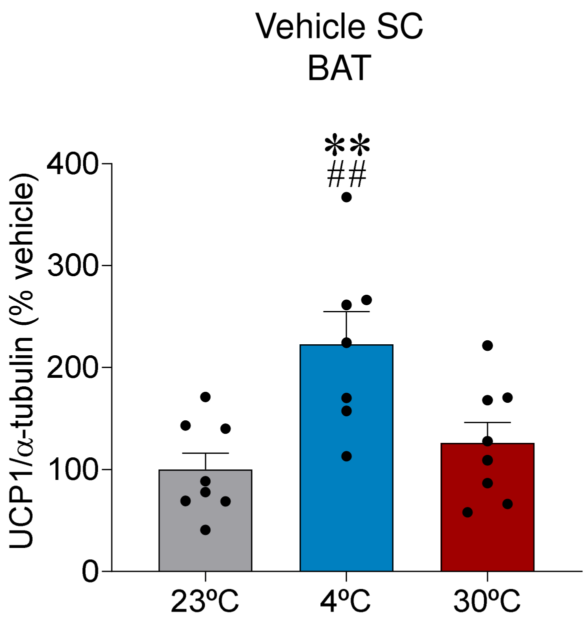

B

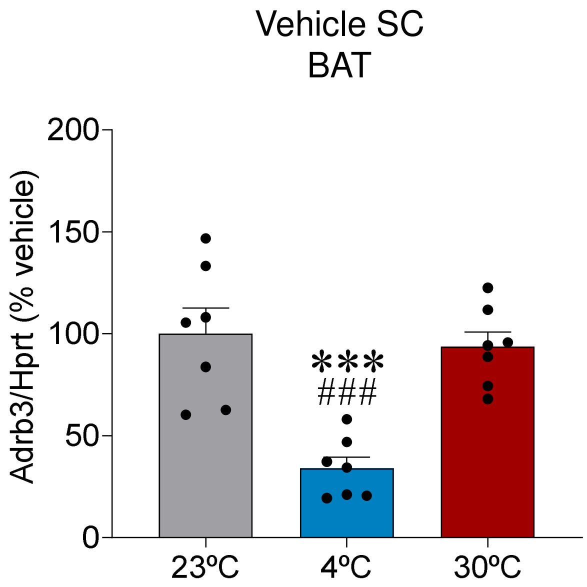

Supplemental Figure 1. Effects of temperature on BAT UCP1 and *Adrb3* in rats treated SC with vehicle

(A) UCP1 protein levels in the BAT and **(B)** *Adrb3* mRNA levels in the BAT of euthyroid rats (peripherally treated (SC) with vehicle). Data are represented as MEAN±SEM. n=7-8 rats/group. Statistical significance was determined by ANOVA. **P<0.01, ***P<0.001 vs. 23ºC; ##P<0.01, ###P<0.001 4ºC vs. 30ºC.

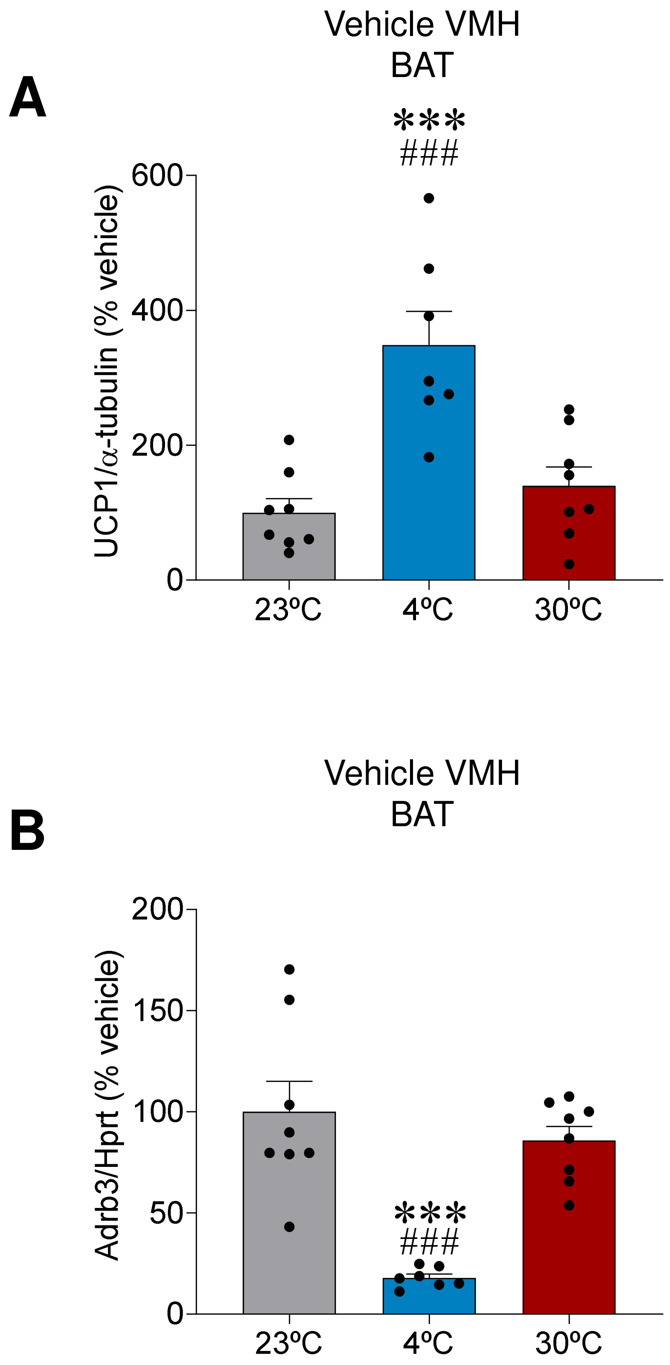

Supplemental Figure 2. Effects of temperature on BAT UCP1 and *Adrb3* in rats stereotaxically treated in the VMH with vehicle

(A) UCP1 protein levels in the BAT and **(B)** *Adrb3* mRNA levels in the BAT of rats stereotaxically treated in the VMH with vehicle. Data are represented as MEAN±SEM. n=7-8 rats/group. Statistical significance was determined by ANOVA. ***P<0.001 vs. 23ºC; ###P<0.001 4ºC vs. 30ºC.
